# Supplementary material for: Tuning of Ag Nanoparticle Properties in Cellulose Nanocrystals/Ag Nanoparticle Hybrid Suspensions by H2O2 Redox Post-Treatment: The Role of the H2O2/AgNP Ratio
Source: Nanomaterials (Basel). 2020 Aug 8;10(8):1559. doi: 10.3390/nano10081559 (PMC7466478; doi:10.3390/nano10081559)
Supplement: Supplementary file 1 [file nanomaterials-10-01559-s001.pdf]

Supplementary Information

Tuning of Ag Nanoparticle Properties in Cellulose Nanocrystals/Ag Nanoparticle Hybrid Suspensions by H_2O_2 Redox Post-Treatment: The Role of the H_2O_2/AgNP Ratio

Figure S1

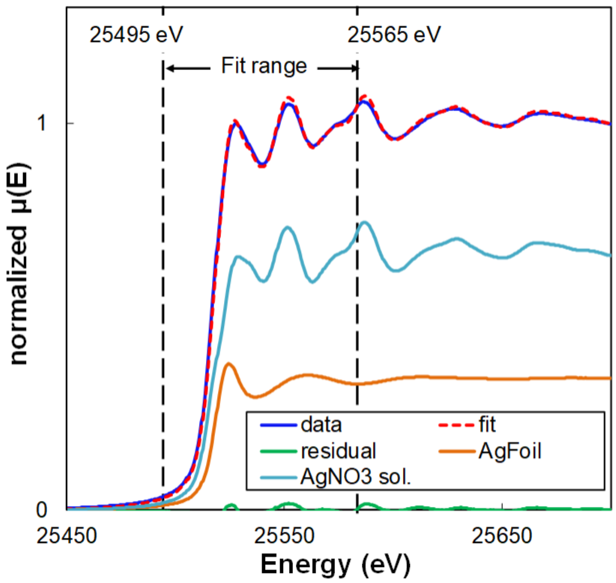

Figure S1. Example of a XANES spectrum of CNC/AgNP hybrid and its corresponding linear combination fit (LCF) using Agfoil and $AgNO_3$ aqueous solution as components.

Table S1. R-factor and Chi-square values for the linear combination fitting procedure applied to the XANES spectra of CNC/AgNP hybrid suspension at 8.7 wt% mixed with various H_2O_2 volumes (i.e., various H_2O_2/AgNP mass ratios, α).

H_2O_2 vol. (μL)	α (H_2O_2/AgNP mass ratio)	R-factor	Chi-square	Ag_0 (%)[1]
**0**	0	0.0007017	0.01885	65 ± 2
**40**	0.07	0.0006697	0.01803	77 ± 2
**80**	0.13	0.0002817	0.00760	82 ± 2
**120**	0.20	-	-	-
**160**	0.27	0.0002519	0.00646	95 ± 3
**250**	0.42	0.0004274	0.01152	94 ± 3

[1] the standard error as 3% of the measured value.

Figure S2

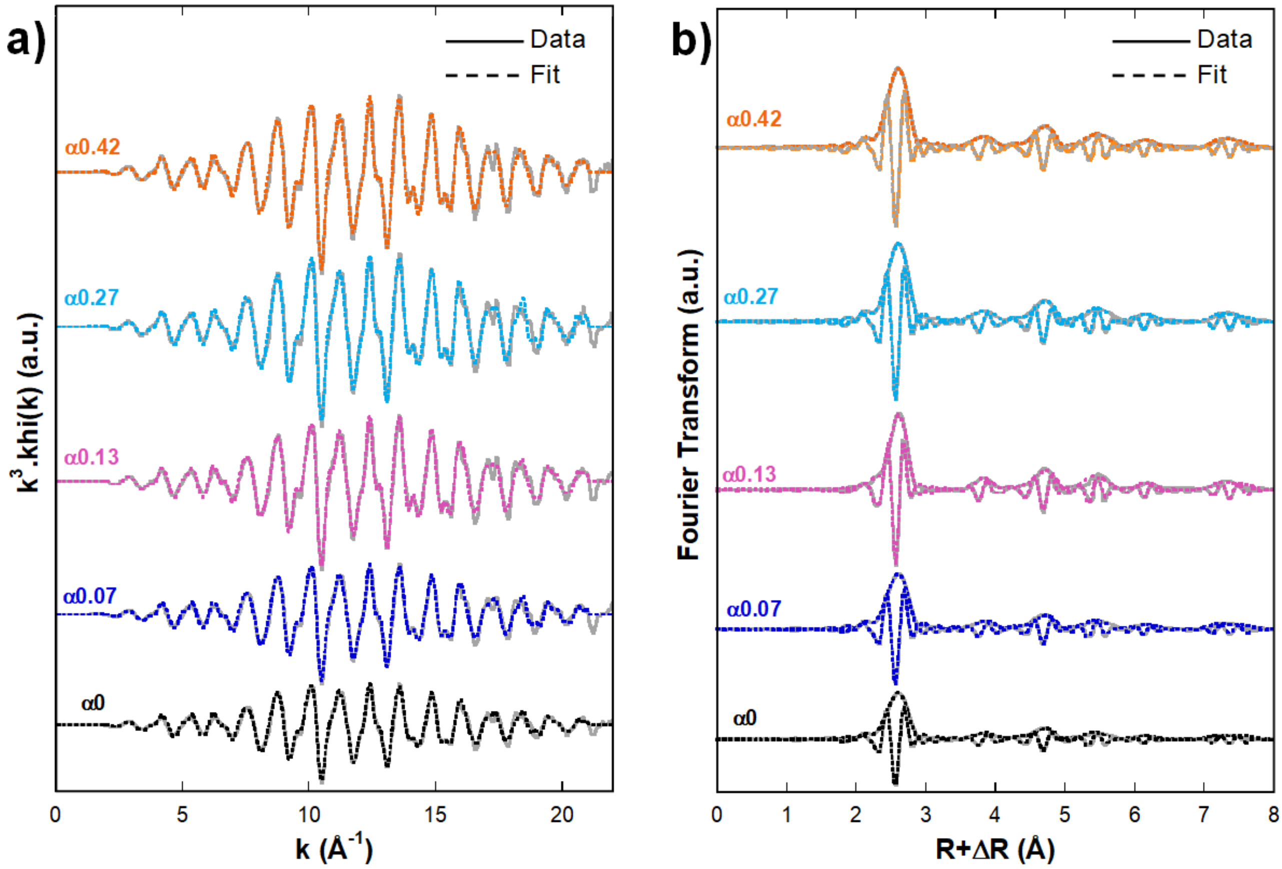

Figure S2. (a) EXAFS spectra Fourier transform (solid gray lines) and fit (dotted lines); (b) magnitude and imaginary part (solid gray lines) and fit (dotted lines) of the Fourier transform; CNC/AgNP hybrid suspensions at 8.7 wt% AgNP treated with various H_2O_2 volumes (i.e., different α values).

Table S2. EXAFS fit results for CNC/AgNP hybrid suspensions at 8.7 wt% AgNP treated with various H_2O_2 volumes (i.e., different α values).

	Degeneracy of the paths					Debye-Waller factor σ^2					Variation in interatomic distance ΔR (Å)					Interatomic distance R (Å)				
	**α0**	**α0.07**	**α0.13**	**α0.27**	**α0.42**	**α0**	**α0.07**	**α0.13**	**α0.27**	**α0.42**	**α0**	**α0.07**	**α0.13**	**α0.27**	**α0.42**	**α0**	**α0.07**	**α0.13**	**α0.27**	**α0.42**
Ag1 ss	7.5 ± 0.4	8.5 ± 0.5	9.5 ± 0.5	11.4 ± 0.6	11.4 ± 0.6	0.0036	0.0034	0.0031	0.0032	0.0032	-0.030	-0.029	-0.028	-0.028	-0.028	2.875	2.877	2.878	2.877	2.878
Ag2 ss	2.2 ± 1.0	2.7 ± 1.2	3.2 ± 1.2	4.2 ± 1.5	4.2 ± 1.5	0.0035	0.0034	0.0029	0.0036	0.0034	-0.037	-0.037	-0.038	-0.038	-0.038	4.072	4.072	4.071	4.071	4.071
Ag1 Ag1 at	48*	48*	48*	48*	48*	0.0054	0.0051	0.0047	0.0049	0.0048	-0.045	-0.043	-0.042	-0.042	-0.042	4.313	4.315	4.316	4.316	4.317
Ag3 ss	22.9 ± 3.4	24.7 ± 3.6	26.7 ± 3.7	28.8 ± 3.8	28.8 ± 3.9	0.0066	0.0061	0.0054	0.0054	0.0053	-0.036	-0.034	-0.034	-0.034	-0.034	4.996	4.999	4.998	4.998	4.998
Ag1 Ag3 ot	96*	96*	96*	96*	96*	0.0051	0.0047	0.0043	0.0043	0.0043	-0.024	-0.023	-0.023	-0.023	-0.022	5.398	5.399	5.399	5.399	5.399
Ag4 ss	5.6 ± 3.9	4.7 ± 4.3	3.8 ± 4.4	3.2 ± 4.2	3.0 ± 4.3	0.0052	0.0047	0.0037	0.0035	0.0033	-0.014	-0.014	-0.015	-0.015	-0.014	5.797	5.797	5.796	5.796	5.797
Ag1 Ag4 fs	24*	24*	24*	24*	24*	0.0088	0.0081	0.0069	0.0067	0.0065	-0.044	-0.043	-0.043	-0.043	-0.042	5.767	5.768	5.768	5.768	5.769
Ag1 Ag1 fta	12*	12*	12*	12*	12*	0.0142	0.0137	0.0125	0.0130	0.0127	-0.061	-0.058	-0.056	-0.057	-0.055	5.750	5.753	5.755	5.754	5.756
Ag1 Ag4 Ag1 dfs	12*	12*	12*	12*	12*	0.0088	0.0081	0.0069	0.0067	0.0065	-0.044	-0.043	-0.043	-0.043	-0.042	5.767	5.768	5.768	5.768	5.769
Ag5 ss	2.7 ± 2.2	3.4 ± 2.5	4.5 ± 2.3	4.9 ± 2.9	6.0 ± 3.4	0.0015	0.0014	0.0010	0.0012	0.0016	-0.058	-0.055	-0.055	-0.055	-0.054	6.439	6.442	6.442	6.442	6.443
Ag7 ss	35.2 ± 8.1	31.5 ± 6.5	29.4 ± 5.7	30.4 ± 6.3	31.3± 6.9	0.0036	0.0026	0.0017	0.0019	0.0019	-0.059	-0.057	-0.054	-0.053	-0.052	7.628	7.630	7.633	7.634	7.635
Ag1 Ag7 ot	96*	96*	96*	96*	96*	0.0036	0.0030	0.0024	0.0026	0.0026	-0.029	-0.027	-0.026	-0.027	-0.026	7.784	7.785	7.786	7.786	7.786
Ag3 Ag7 ot	96*	96*	96*	96*	96*	0.0051	0.0043	0.0035	0.0036	0.0036	-0.035	-0.033	-0.034	-0.034	-0.033	7.777	7.779	7.779	7.779	7.779

	**α0**	**α0.07**	**α0.13**	**α0.27**	**α0.42**
R-factor	0.015	0.014	0.012	0.010	0.011
ΔE_0	0.00 ± 0.61	0.27 ± 0.60	0.31 ± 0.60	0.17 ± 0.54	0.08 ± 0.57

ss: single scattering; at: acute triangle; ot: obtuse triangle; fs: forward scattering; dfs: double forward scattering; fta: forward through absorber. Fixed parameters are indicated by a "*". The amplitude reduction factor S_0^2 was fixed at 0.978 Å. Errors obtained for σ^2 were systematically lower than 0.0020; errors obtained for ΔR and R were systematically lower than 0.0094.

Figure S3

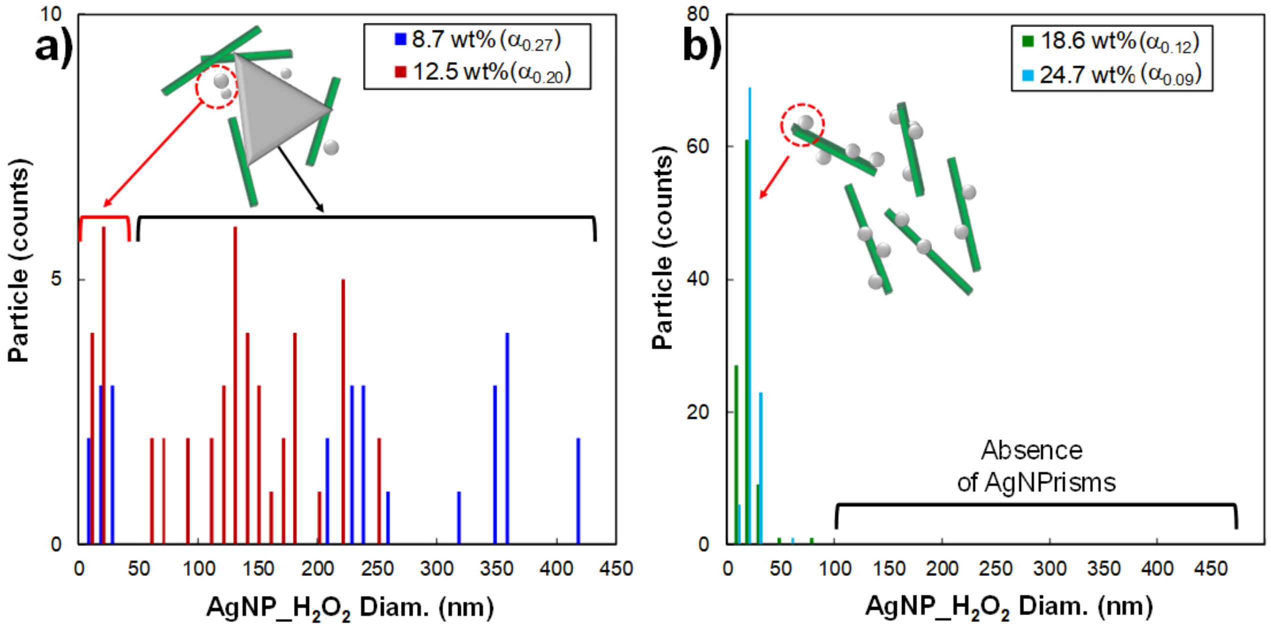

Figure S3. AgNP size distributions of CNC/AgNP_H_2O_2 hybrids at (a) 8.7 wt% and 12.5 wt% AgNP and (b) 18.6 wt% and 24.7 wt% AgNP, mixed with the addition of 160 µL of H_2O_2, thus varying α from 0.09 to 0.27.

Table S3. Average diameter of AgNPs_H_2O_2 in CNC/AgNP hybrids at 8.7 wt%, 12.5 wt%, 18.6 wt% and 24.7 wt% AgNP, mixed with the addition of 160 µL of H_2O_2 to reach various α values.

AgNP (wt%)	α	NP shape	Avg diam. (nm)	AgNP count
**8.7**	0.27	AgNPrisms	296.1 ± 70.0	20
**12.5**	0.20	AgNPrisms	144.8 ± 51.2	40
**18.6**	0.12	Spherical AgNPs	14.2 ± 8.8	100
**24.7**	0.09	Spherical AgNPs	16.8 ± 6.2	100

Figure S4

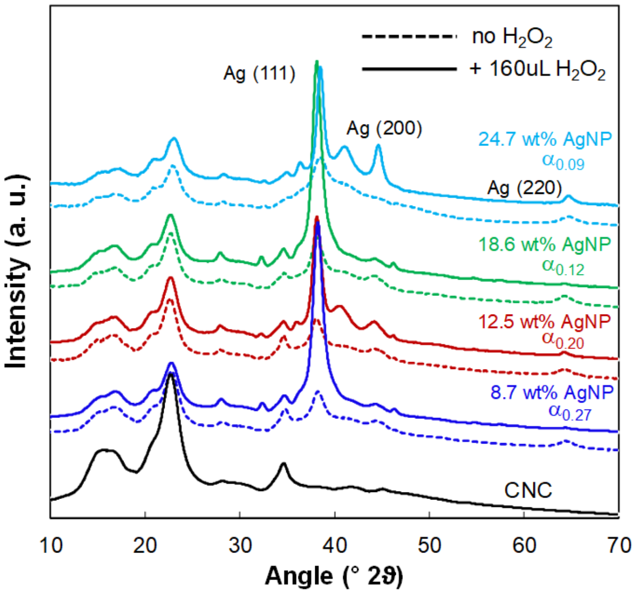

Figure S4. XRD diffractograms of hybrids at different initial AgNP content treated with 160 µL of H_2O_2 (i.e., α values above and below critical value of 0.20).

Table S4. R-factor and Chi-square values for the linear combination fitting procedure applied to the XANES region of CNC/AgNP hybrid suspension at various AgNP contents mixed with 160 µL H_2O_2.

Initial AgNP (wt%)	α (H_2O_2/AgNP mass ratio)	R-factor	Chi-square	Ag_0 (%)[1]
**8.7**	0.27	0.0002519	0.00646	95 ± 3
**12.5**	0.20	0.0003107	0.00840	97 ± 3
**18.6**	0.12	0.0003021	0.00830	50 ± 3
**24.7**	0.09	0.0006323	0.01744	29 ± 3

[1] the standard error as 3% of the measured value

Figure S5

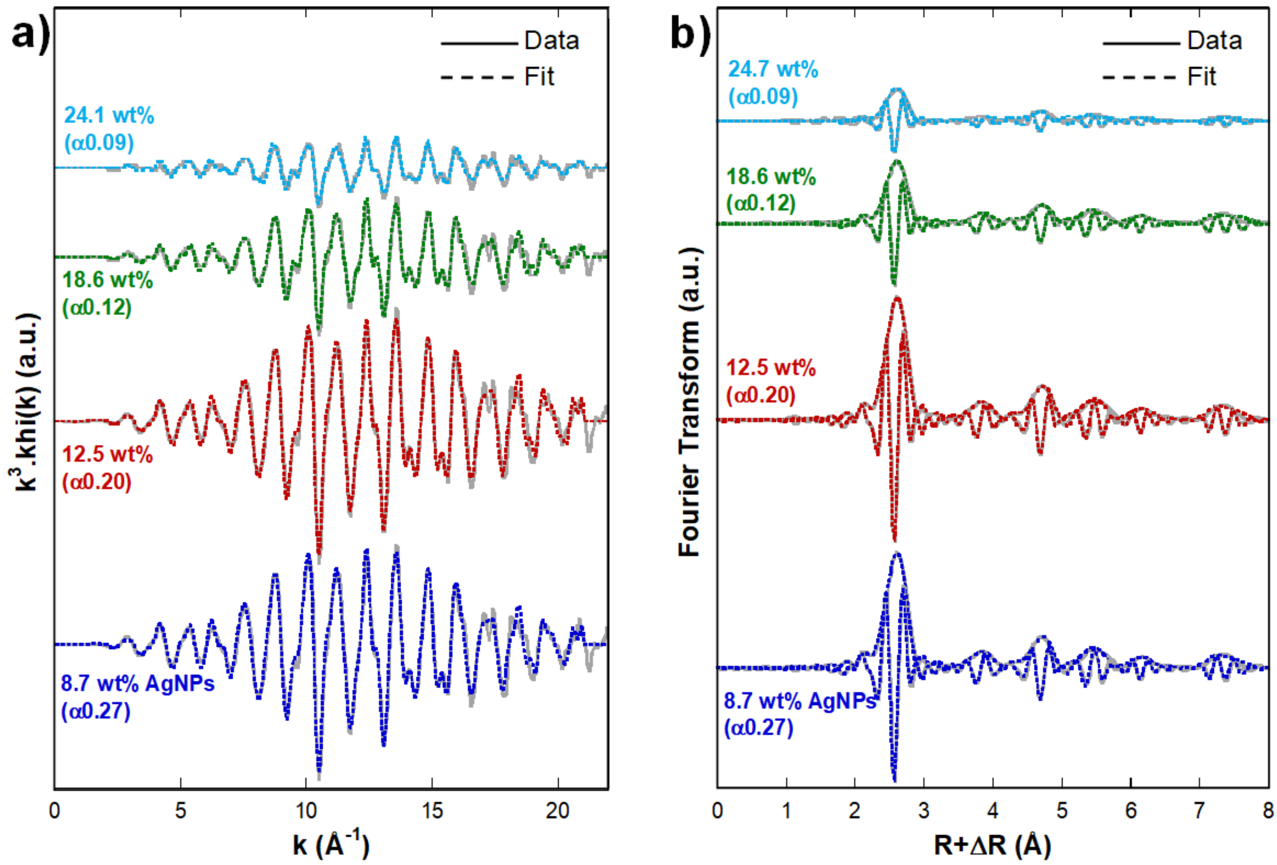

Figure S5. (a) EXAFS spectra Fourier transform (solid gray lines) and fit (dotted lines); (b) magnitude and imaginary part (solid gray lines) and fit (dotted lines) of the Fourier transform of CNC/AgNP hybrid suspensions at various AgNP contents treated with 160 μL of H_2O_2 (i.e., α values from 0.09 to 0.27).

Table S5. EXAFS fit results for CNC/AgNP hybrid suspensions at CNC/AgNP_H_2O_2 hybrids at various AgNP_H_2O_2 contents, treated with 160 µL of H_2O_2 (i.e., α above and below the critical value of 0.20).

	**Degeneracy of the paths**				**Debye-Waller factor σ²**				**Variation in interatomic distance ΔR (Å)**				**Interatomic distance R (Å)**			
	8.7% α0.27	12.5% α0.20	18.6% α0.12	24.1% α 0.09	8.7% α0.27	12.5% α0.20	18.6% α0.12	24.1% α 0.09	8.7% α0.27	12.5% α0.20	18.6% α0.12	24.1% α 0.09	8.7% α0.27	12.5% α0.20	18.6% α0.12	24.1% α 0.09
Ag1 ss	11.4 ± 0.6	11.1 ± 0.5	5. 7 ± 0.4	3.1 ± 0.3	0.0033	0.0030	0.0030	0.0032	-0.029	-0.028	-0.029	-0.030	2.876	2.878	2.877	2.876
Ag2 ss	4.2 ± 1.5	4.1 ± 1.3	1.7 ± 0.9	0.3 ± 0.3	0.0023	0.0030	0.0024	0.0001	-0.037	-0.039	-0.036	-0.040	4.072	4.070	4.073	4.069
Ag1 Ag1 at	48*	48*	48*	48*	0.0050	0.0044	0.0045	0.0048	-0.044	-0.041	-0.044	-0.022	4.314	4.317	4.315	4.336
Ag3 ss	28.8 ± 3.8	29.2 ± 3.7	22.0 ± 3.4	16.8 ± 3.0	0.0059	0.0052	0.0059	0.0069	-0.035	-0.034	-0.034	-0.035	4.997	4.999	4.997	4.997
Ag1 Ag3 ot	96*	96*	96*	96*	0.0046	0.0041	0.0045	0.0051	-0.023	-0.022	-0.023	-0.024	5.398	5.400	5.399	5.398
Ag4 ss	3.2 ± 4.2	3.7 ± 4.3	9.8 ± 2.4	17.2 ± 2.1	0.0036	0.0034	0.0038	0.0050	-0.014	-0.015	0.001	0.035	5.797	5.796	5.812	5.846
Ag1 Ag4 fs	24*	24*	24*	24*	0.0069	0.0064	0.0067	0.0082	-0.043	-0.043	-0.028	0.006	5.768	5.768	5.783	5.817
Ag1 Ag1 fta	12*	12*	12*	12*	0.0133	0.0118	0.0119	0.0128	-0.059	-0.055	-0.058	-0.059	5.752	5.756	5.753	5.752
Ag1 Ag4 Ag1 dfs	12*	12*	12*	12*	0.0069	0.0064	0.0067	0.0082	-0.043	-0.043	-0.028	0.006	5.768	5.768	5.783	5.817
Ag5 ss	4.9 ± 2.9	5.2 ± 2.9	3.4 ± 2.3	2.0 ± 2.0	0.0020	0.0012	0.0011	0.0016	-0.057	-0.053	-0.056	-0.053	6.440	6.444	6.441	6.444
Ag7 ss	30.4 ± 6.3	29.5 ± 5.6	31.8 ± 6.4	88.6 ± 37.9	0.0027	0.0014	0.0023	0.0116	-0.055	-0.053	-0.056	-0.044	7.633	7.634	7.631	7.643
Ag1 Ag7 ot	96*	96*	96*	96*	0.0030	0.0022	0.0027	0.0074	-0.028	-0.026	-0.027	-0.027	7.785	7.787	7.785	7.785
Ag3 Ag7 ot	96*	96*	96*	96*	0.0043	0.0033	0.0041	0.0093	-0.034	-0.033	-0.033	-0.034	7.778	7.780	7.779	7.779

	**8.7% α0.27**	**12.5% α0.20**	**18.6% α0.12**	**24.1% α 0.09**
R-factor	0.010	0.009	0.022	0.048
ΔE_0	-0.17 ± 0.54	0.52 ± 0.44	0.17 ± 0.65	0.07 ± 0.95

ss: single scattering; at: acute triangle, ot: obtuse triangle; fs: forward scattering; dfs: double forward scattering; fta: forward through absorber. Fixed parameters are indicated by a "*". The amplitude reduction factor S_0^2 was fixed at 0.978 Å. Errors obtained for σ² were systematically lower than 0.032; errors obtained for ΔR and R were systematically lower than 0.016.

Figure S6

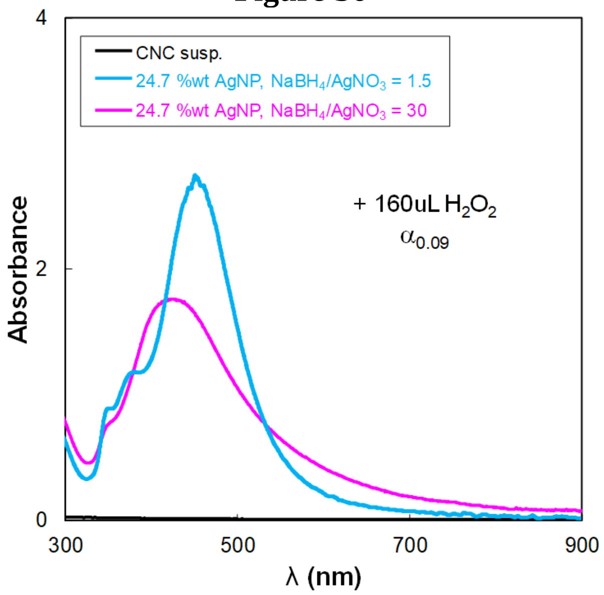

Figure S6. UV-Vis spectra of CNC/AgNP hybrid suspension prepared at 24.7 %wt at two different $NaBH_4/AgNO_3$ molar ratios (i.e., 1.5 and 30) and then mixed with 160 μL H_2O_2 (i.e., α= 0.09). In our experimental conditions, such a parameter did not affect the H_2O_2 redox post-treatment.
